# Chronic spontaneous cholinergic urticaria: advances in immunological mechanisms focusing on neuro-immune crosstalk and novel targeted therapeutic strategies

**DOI:** 10.3389/fimmu.2026.1848638

**Published:** 2026-06-05

**Authors:** Meilv Yang, Suxian Lin, Yang Lu, Huanzhi Jin, Xueliang Zhang, Zheyan Chen

**Affiliations:** 1 Rheumatology and Immunology Department, Wenzhou Third Clinical Institute Affiliated to Wenzhou Medical University, The Third Affiliated Hospital of Shanghai University, Wenzhou People’s Hospital, Wenzhou, China; 2 Department of General Practice, Wenzhou Third Clinical Institute Affiliated to Wenzhou Medical University, The Third Affiliated Hospital of Shanghai University, Wenzhou People’s Hospital, Wenzhou, China; 3 Department of Plastic and Reconstructive Surgery, Wenzhou Third Clinical Institute Affiliated to Wenzhou Medical University, The Third Affiliated Hospital of Shanghai University, Wenzhou People’s Hospital, The Wenzhou People’s Hospital Affiliated of Hangzhou Medical College, Wenzhou, China

**Keywords:** autoimmunity, chronic spontaneous cholinergic urticaria, cytokines, mast cells, neuro-immune crosstalk, targeted therapy

## Abstract

Chronic spontaneous cholinergic urticaria (CSU-Chol) is a chronic inflammatory skin disorder characterized by intense pruritus and small wheals triggered by a rise in core body temperature. This review aims to systematically elucidate its complex pathogenesis, with a particular focus on the pivotal role of neuro-immune crosstalk in disease initiation and progression. We will provide an in-depth analysis of key immunological components, including aberrant mast cell activation, autoimmune responses, and dysregulation of the cytokine network. Furthermore, the review will detail how neuropeptides released from sensory nerve endings engage in dialogue with the immune system. Building on this mechanistic foundation, the article will further delineate the evolution of treatment strategies, from traditional antihistamines to emerging targeted therapies aimed at pathways involving IgE, IL-4/IL-13, and mast cell signaling. The overarching goal is to consolidate current knowledge to provide a theoretical basis for precise clinical diagnosis and management, while highlighting promising future research directions for this challenging condition.

## 1 Introduction

Chronic spontaneous cholinergic urticaria represents a significant and often challenging subtype within the spectrum of chronic inducible urticaria (CIndU). Chronic urticaria (CU) itself is defined by the presence of intensely pruritic wheals, with individual lesions resolving within 24 hours, persisting for a duration of at least 6 weeks (1). It is broadly categorized into chronic spontaneous urticaria (CSU), which occurs without clearly identifiable triggers, and CIndU, where specific triggers such as dermatographism, heat (cholinergic), cold, exercise, delayed pressure, or solar exposure provoke symptoms (1). Cholinergic urticaria (CholU), triggered by a rise in core body temperature from exercise, emotional stress, or hot baths, is a well-recognized form of CIndU.The most significant difference between CholU and CSU lies in the presence or absence of specific triggering factors. Epidemiological studies highlight its clinical relevance; in a Japanese primary care survey, CholU accounted for 5.7% of all urticaria cases, showing a particular predilection for young males in their 10s-20s (2). Similarly, a Turkish pediatric study found CholU in 4.8% of children with chronic inducible urticaria (3). The clinical burden is substantial, as patients with concomitant CSU and CIndU, including CholU, experience longer times to control symptoms and poorer disease control as measured by tools like the Urticaria Control Test (2, 4). This overlap is not uncommon, with studies reporting that 21-35% of patients with CIndU may also have CSU, a combination associated with a more severe and refractory disease course, including higher frequencies of angioedema and emergency referrals (5, 6). The diagnosis of CholU, like other CIndU subtypes, can be subject to significant delays, with registry data indicating a median diagnostic delay of 4 months for CholU patients, longer than for many other forms (5). This underscores the need for greater awareness of its clinical presentation and diagnostic work-up. In this review, we will discuss current understanding of CholU and CSU and how similarities and differences in the pathophysiology may impact presentation of overlapping conditions like chronic spontaneous cholinergic urticaria.

The pathogenesis of chronic spontaneous cholinergic urticaria extends beyond a simple cholinergic response, implicating a complex interplay between neural activation and immune dysregulation. While the physical trigger of increased body temperature is central, the resultant whealing and pruritus are mediated through mast cell activation and histamine release. However, emerging evidence suggests this is an oversimplification. Research indicates that patients with different CIndU subtypes, including CholU, exhibit distinct immunological profiles compared to those with standalone CSU. For instance, patients with CholU, along with those with symptomatic dermographism (SD) and cold urticaria (ColdU), have been shown to have lower levels of C-reactive protein (CRP), higher total serum immunoglobulin E (IgE) levels, and higher basophil counts relative to CSU patients (7). These findings point towards underlying heterogeneity and potentially different endotypic pathways. The concept of “autoimmune” or “autoallergic” mechanisms, well-described in CSU, may also play a role in a subset of CholU cases. Type I autoimmune (autoallergic) CSU is driven by IgE antibodies directed against autoallergens, while type IIb involves mast cell-targeted autoantibodies (e.g., IgG anti-FcϵRI or anti-IgE) (8). It is plausible that similar autoantibodies could lower the activation threshold of mast cells in CholU, making them more susceptible to neurogenic stimuli. The physical stimulus of heat likely activates cutaneous sensory nerve fibers, leading to the release of neuropeptides such as substance P and vasoactive intestinal peptide. These neuropeptides can directly induce mast cell degranulation and vasodilation, creating a bidirectional neuro-immune loop that perpetuates inflammation and symptom chronicity. This intricate crosstalk forms the pathological basis for the sustained disease activity observed in many patients.

The limitations of conventional therapy have been a major driver for investigating these deeper immunopathological mechanisms. First-line treatment for all forms of CU, including CholU, involves second-generation H1-antihistamines (sgAHs). However, response rates in CholU are often suboptimal. A retrospective analysis of treatment responses in CIndU revealed that while the overall antihistamine response rate was 78.3% in isolated CIndU, it was significantly lower in CholU (60.9%) compared to SD (83.2%) and ColdU (78.3%) (9). Furthermore, patients with CholU who are refractory to antihistamines often have distinct clinical features, including a longer disease duration, the presence of angioedema, concomitant CSU, or mixed CIndU subtypes (e.g., having both CholU and another form like SD) (9). The presence of mixed CIndU is clinically relevant; for example, combinations like SD and CholU occur in a notable proportion of patients and are associated with higher rates of comorbid CSU and angioedema (10). For patients unresponsive to standard or updosed antihistamines, the anti-IgE monoclonal antibody omalizumab represents an effective second-line option. Omalizumab has demonstrated efficacy across various CU subtypes. In CIndU, overall response rates to omalizumab can be as high as 86.5% (9). Case reports also support its use in complex, overlapping conditions, such as a patient with simultaneous CSU, angioedema, and CholU, where omalizumab improved urticaria control (11). Nevertheless, some patients remain refractory even to omalizumab, highlighting the need for novel therapeutic agents that target alternative pathways implicated in mast cell activation and neuro-immune communication.

This evolving understanding of pathogenesis has catalyzed the development of novel targeted therapies aimed at specific immune and neural pathways. The investigational drug lirentelimab, an anti-sialic acid-binding immunoglobulin-like lectin 8 (Siglec-8) monoclonal antibody, exemplifies this new direction. Siglec-8 is an inhibitory receptor expressed on mast cells and eosinophils. In a proof-of-concept phase 2a study involving patients with antihistamine-refractory CU, lirentelimab showed promising activity. Among a cohort of 11 patients with CholU, 100% (7 of 7 evaluable patients) had a negative response to an exercise provocation test (Pulse-Controlled Ergometry) after treatment, and 82% achieved a complete response based on Urticaria Control Test scores (12). Unfortunately, the drug’s clinical trials were declared unsuccessful in phase 2b, and further research is still required for this pathway. Other potential future treatments include drugs that inhibit intracellular mast cell activation pathways (e.g., Bruton’s tyrosine kinase inhibitors) or agents that silence mast cells by binding to other inhibitory receptors (8). Furthermore, the success of biologics like dupilumab, which targets the IL-4/13 receptor, in related conditions hints at the potential role of type 2 inflammation. In a reported case, dupilumab induced complete remission of CSU with angioedema and provided satisfactory control of cholinergic flare-ups in a patient with overlapping urticaria and atopic dermatitis (11). This underscores the therapeutic potential of modulating broader cytokine networks involved in mast cell priming and neuroimmune signaling. The ongoing refinement of patient-reported outcome measures, such as the Cholinergic Urticaria Activity Score and the Cholinergic Urticaria Quality of Life Questionnaire, will be crucial for accurately assessing the efficacy of these emerging therapies in clinical trials and practice (8).

## 2 Clinical features and diagnostic challenges of chronic spontaneous cholinergic urticaria

### 2.1 Unique provocation patterns and clinical manifestations

Cholinergic urticaria (CholU) is a subtype of chronic inducible urticaria (CIndU) characterized by the development of small, intensely pruritic wheals triggered by a rise in core body temperature, such as during exercise, passive warming, hot baths, or emotional stress (13). The typical lesions are described as pinpoint-sized (1–3 mm in diameter), monomorphic, and surrounded by a significant erythematous flare (14). These wheals and the associated severe itching typically appear within 2–30 minutes of provocation and resolve spontaneously within 30–90 minutes, though the intense pruritus can significantly impair quality of life (14). A critical clinical feature is the frequent co-existence of CholU with chronic spontaneous urticaria (CSU), which adds complexity to diagnosis and management. Epidemiological studies consistently report this overlap; for instance, a large international registry study found that 21% of chronic urticaria patients had a combination of CSU and CIndU (5). Another study confirmed the concomitance of CIndU and CSU in 35.3% of patients, noting a higher frequency of cholinergic urticaria within this group (4). This overlap is further highlighted in a comparative analysis where approximately 30% of CIndU patients had concomitant CSU, and these patients with combined disease (CSU + CIndU) presented with a more severe and refractory clinical course, including more frequent angioedema and lower disease control scores (6). Therefore, confirming the diagnosis of CholU requires a careful history and standardized provocation testing, such as exercise challenge (e.g., pulse-controlled ergometry) or passive warming (e.g., hotbath), to objectively reproduce the characteristic wheals (13, 15). The clinical presentation can vary, with some patients experiencing not only wheals but also transient dermal pain upon sweating, and a subset may have associated hypohidrosis or anhidrosis, further defining specific disease phenotypes (16, 17).

### 2.2 Limitations of current diagnostic criteria and exploration of biomarkers

The diagnosis of cholinergic urticaria primarily relies on a detailed clinical history and confirmatory provocation tests, as there is no objective laboratory gold standard, making it susceptible to misdiagnosis or confusion with other forms of urticaria or pruritic disorders (1). This diagnostic uncertainty is reflected in data showing significant diagnostic delays, particularly for CIndU subtypes like CholU. One registry-based analysis reported that the diagnosis of chronic urticaria was delayed by at least one year in 24% of patients, with patients having symptomatic dermographism or cholinergic urticaria experiencing the longest delays (median of 4 months) compared to those with standalone CSU (5). While the detection of specific autoantibodies, such as anti-FcϵRIα or anti-IgE, is valuable for identifying autoimmune endotypes in chronic spontaneous urticaria, their specific role and utility in CholU remain to be clearly defined (8). Current research is actively exploring more objective biomarkers to achieve a more precise diagnosis. These include analyzing inflammatory mediators in sweat, measuring changes in blood histamine or cytokine levels following provocation, and detecting neuropeptides (17). For example, studies have investigated the autologous sweat skin test (ASwST) and IgE sensitization to sweat components. Research indicates that a subset of CholU patients shows a positive ASwST, suggesting a “sweat allergy” or hypersensitivity mechanism, though prevalence rates vary geographically (18, 19). Furthermore, IgE against specific antigens like MGL_1304 from *Malassezia globosa* has been identified as a potential sweat allergen, and the detection of anti-MGL_1304 IgE in serum shows promise as a diagnostic tool for type I sweat allergy with good sensitivity and specificity (20). Other explored biomarkers involve assessing sweat gland function and composition; patients with CholU often exhibit altered sweating, and analysis of sweat electrolyte composition (e.g., increased potassium concentration) and tight junction protein expression in sweat glands (e.g., altered claudin-3 and occludin distribution) may provide insights into underlying pathomechanisms (21). Additionally, the validation of patient-reported outcome measures like the Cholinergic Urticaria Activity Score (CholUAS7) offers a standardized way to quantify disease activity, which, while not a diagnostic biomarker, is crucial for assessing severity and treatment response in clinical practice and trials (22).

## 3 The core role of mast cells and mechanisms of abnormal activation

### 3.1 Autoantibody-mediated mast cell activation

In chronic spontaneous urticaria, based on differences in pathogenesis, it can be divided into two endotypes: type I, which is primarily autoallergic, and type IIb, which is primarily autoimmune. Type I autoallergy involve an IgE-mediated response to autoantigens, inducing the release of histamine and vasoactive mediators by mast cells/basophils. The primary manifestation is the erroneous recognition of self-tissues as allergens, leading to the production of specific IgE antibodies. Known autoallergens such as TPO and IL-24 may be captured by antigen-presenting cells, resulting in the activation of Th2 cells. These Th2 cells promote the production of auto-reactive IgE antibodies by plasma cells (23). The IgE autoantibodies bind to tissue-resident mast cells, triggering their activation and subsequent degranulation (23). Type IIb refers to the response of IgG autoantibodies against the IgE receptor (FcϵRI) or against IgE itself (24). By cross-linking these receptors on the surface of effector cells, these IgG autoantibodies mimic the action of allergens in IgE-mediated allergies, leading to mast cell and basophil degranulation and the release of inflammatory mediators such as histamine (25, 26). This autoimmune activation is independent of external allergens, providing a critical explanation for the spontaneous and recurrent nature of wheals and angioedema in CSU and forming a key basis for the chronicity of the disease (27). The type IIb autoimmune CSU is estimated to be present in a subset of patients, with strict diagnostic criteria (including positive autologous serum skin test, immunoassays for IgG autoantibodies, and basophil activation tests) identifying it in less than 10% of CSU cases (28). Detection methods for these functional autoantibodies continue to evolve, with assays like the amplified luminescence proximity homogeneous assay by cross-linking (AlphaCL) being developed to specifically identify autoantibodies capable of cross-linking FcϵRI or IgE molecules (29). The clinical relevance of this autoantibody-mediated pathway is substantial, as patients with this autoimmune endotype often present with more severe disease, a higher prevalence of concomitant autoimmune conditions like autoimmune thyroid disease, and may exhibit a poorer response to first-line antihistamines and even to the anti-IgE therapy omalizumab (28, 30). In contrast, they may show a better response to immunosuppressants like cyclosporine (28, 31). The coexistence of this IgG-mediated (type IIb) autoimmunity with IgE-mediated autoallergy (type I), where IgE autoantibodies target autoantigens, is also recognized, adding complexity to the disease pathogenesis (32, 33). Elevated body temperature, a common trigger in cholinergic urticaria, is hypothesized to potentially enhance this autoantibody-mediated activation, possibly by altering antibody-antigen binding affinity or affecting mast cell membrane fluidity, thereby lowering the threshold for degranulation (30). This interplay underscores the importance of the autoimmune component in driving mast cell activation in a substantial cohort of CSU patients, guiding both diagnostic stratification and therapeutic choices towards more personalized management strategies (34, 35).

### 3.2 Non-antibody-dependent activation pathways

**Figure 1:**
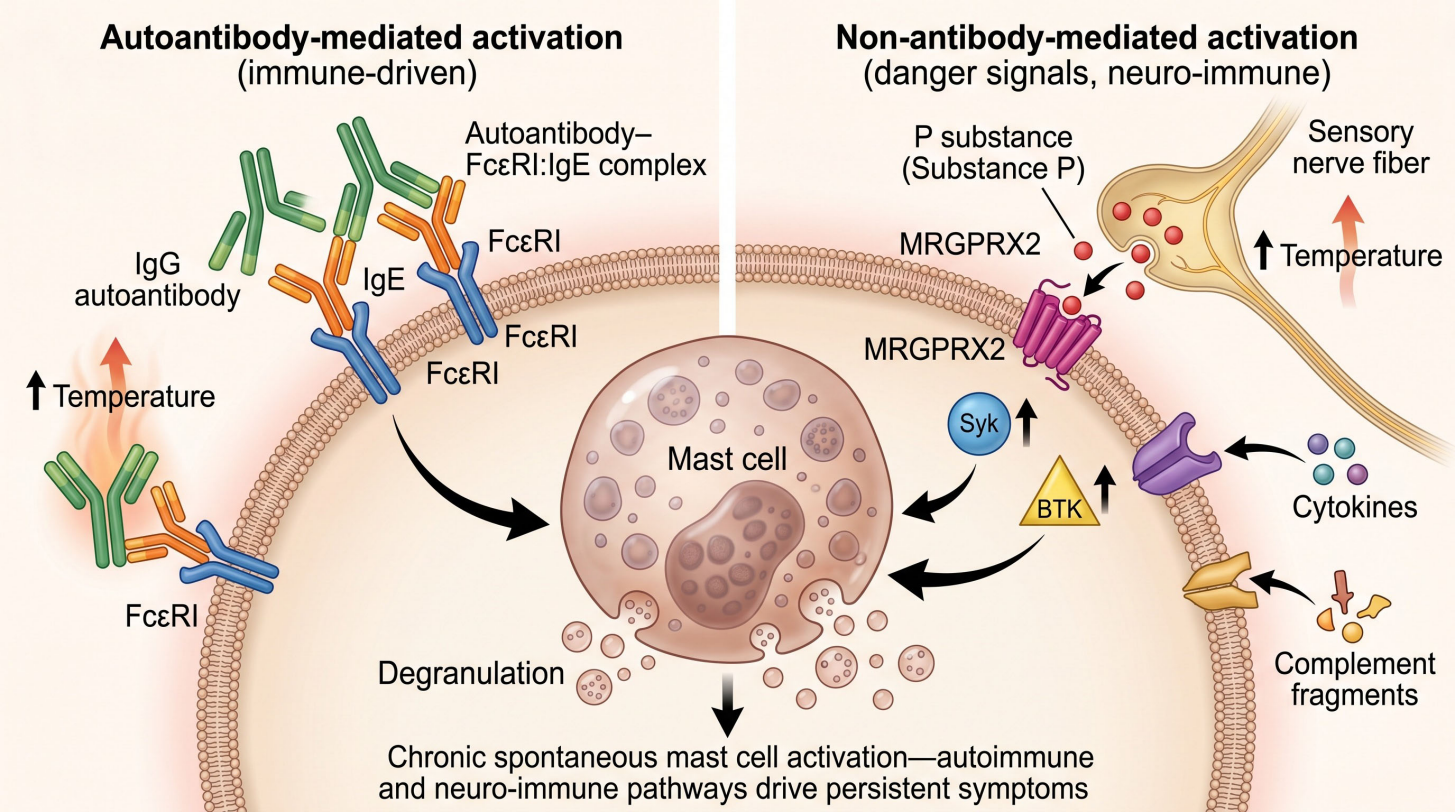
Mechanism of mast cell activation: antibody-mediated activation and non-antibody-mediated activation.

Beyond autoantibody-driven mechanisms, mast cells in chronic spontaneous urticaria (CSU) can be activated through several non-IgE and non-IgG dependent pathways, involving a variety of endogenous danger signals and intrinsic cellular dysregulation (23). These alternative activation routes contribute significantly to the disease’s pathogenesis, particularly in cases where autoimmune markers are absent. A key pathway involves the Mas-related G protein-coupled receptor X2 (MRGPRX2), which is expressed on mast cells and can be activated by a diverse range of endogenous neuropeptides (like substance P) and cationic molecules, as well as certain drugs, leading to degranulation (24). Substance P, released from sensory nerve endings, is a potent activator of mast cells via MRGPRX2 and represents a critical link in the neuro-immune interaction within the skin (36). The release of such neuropeptides can be influenced by various factors, including stress and physical triggers like heat, which may explain symptom exacerbation in certain contexts. Furthermore, other endogenous signals such as complement fragments (e.g., C5a) and certain cytokines can directly or indirectly provoke mast cell activation (27). Internally, dysregulation of intracellular signaling pathways within mast cells and basophils is a proposed central mechanism in CSU pathogenesis (23). Key signaling molecules like spleen tyrosine kinase (Syk) and Bruton’s tyrosine kinase (BTK) are often found in a hyperactive state, lowering the activation threshold of these cells and making them more susceptible to degranulation by a wider array of stimuli (34). This intrinsic hyperreactivity means that mast cells may degranulate more readily in response to lower levels of stimulation. Evidence for this includes the efficacy of novel BTK inhibitors, such as remibrutinib, and fenebrutinib, which are under investigation for CSU as they inhibit these critical signaling pathways downstream of various activation receptors, thereby suppressing mast cell and basophil degranulation (28, 37). Remibrutinib has been shown to inhibit the activation of human basophils and mast cells induced *in vitro* by exposure to serum from CSU patients, independent of their clinical response to omalizumab, highlighting the relevance of this intracellular pathway (37). Its efficacy has been demonstrated in CSU patients and it has received approval from the U.S. FDA. Additionally, other inflammatory cells and their mediators play a modulatory role. Crosstalk between mast cells and infiltrating immune cells like T cells, eosinophils, and basophils in the perivascular skin lesions can further regulate mast cell function (38). For instance, T helper 2 (Th2) cytokines and factors from eosinophils that initiate the extrinsic coagulation pathway can exert powerful influences on mast cell activity (38). The coagulation system itself is implicated, with markers like D-dimer often elevated in CSU, suggesting that thrombin generation and other coagulation factors may also participate in mast cell activation (39). This complex network of non-antibody dependent pathways, involving neuro-immune interactions, complement, coagulation, and intrinsic cellular hyperreactivity, ensures that mast cell activation in CSU can occur through multiple redundant mechanisms, contributing to the heterogeneity of the disease and the variable response to therapies that target only specific pathways like IgE (30, 35) (**Figure 1**).

## 4 Construction and function of neuro-immune interaction networks

### 4.1 The immunomodulatory role of sensory nerve endings and neuropeptides

The intricate neuroimmune crosstalk in chronic spontaneous urticaria (CSU) is fundamentally driven by sensory nerve endings, particularly unmyelinated C-fibers, which express various receptor-type ion channels, including the temperature-sensitive transient receptor potential vanilloid 1 (TRPV1) channel (40). Activation of these channels by elevated core body temperature, a hallmark trigger in cholinergic urticaria, leads to neuronal excitation and the subsequent release of neuropeptides such as substance P (SP) and calcitonin gene-related peptide (CGRP) from sensory nerve terminals (41). These neuropeptides act as crucial immunomodulators, initiating a local “neurogenic inflammation” cascade. Current evidence suggests that the biological activity of SP can be exerted not only through the conventional NK-1 receptor but also through the Mas-related G protein-coupled receptors. CGRP, a receptor highly expressed on skin MCs, triggers non-IgE-mediated degranulation and the release of pro-inflammatory mediators like histamine and tryptase (42, 43). Injecting SP subcutaneously into mice induces MC degranulation, histamine release, and an inflammatory response, simulating CU symptoms in humans (44). Concurrently, released neuropeptides exert direct effects on vascular endothelial cells, causing vasodilation and plasma extravasation, which manifest as wheals and redness (45). Furthermore, these neuropeptides can recruit and activate other immune cells, including eosinophils and T cells, thereby amplifying the local inflammatory milieu (42). For instance, eosinophil-derived proteins like major basic protein can themselves act as agonists for MRGPRX2, creating a positive feedback loop that sustains inflammation (43). This neurogenic circuit is not isolated; it is integrated into a broader senso-immunological framework where nociceptive signals from primary sensory neurons directly modulate peripheral immune function (40). The critical role of this axis is further highlighted by therapeutic interventions; omalizumab treatment in CSU patients has been shown to significantly alter serum levels of SP and CGRP, suggesting that its efficacy may partly stem from modulating this neuroimmune dialogue beyond mere IgE receptor blockade (46). Thus, the activation of temperature-sensitive channels on sensory nerves and the subsequent release of neuropeptides serve as a pivotal initiating event, directly linking physiological triggers like warmth to the immunopathological processes of mast cell degranulation, vascular leakage, and leukocyte recruitment that characterize CSU lesions.

### 4.2 Feedback regulation of neural function by immune cells

**Figure 2:**
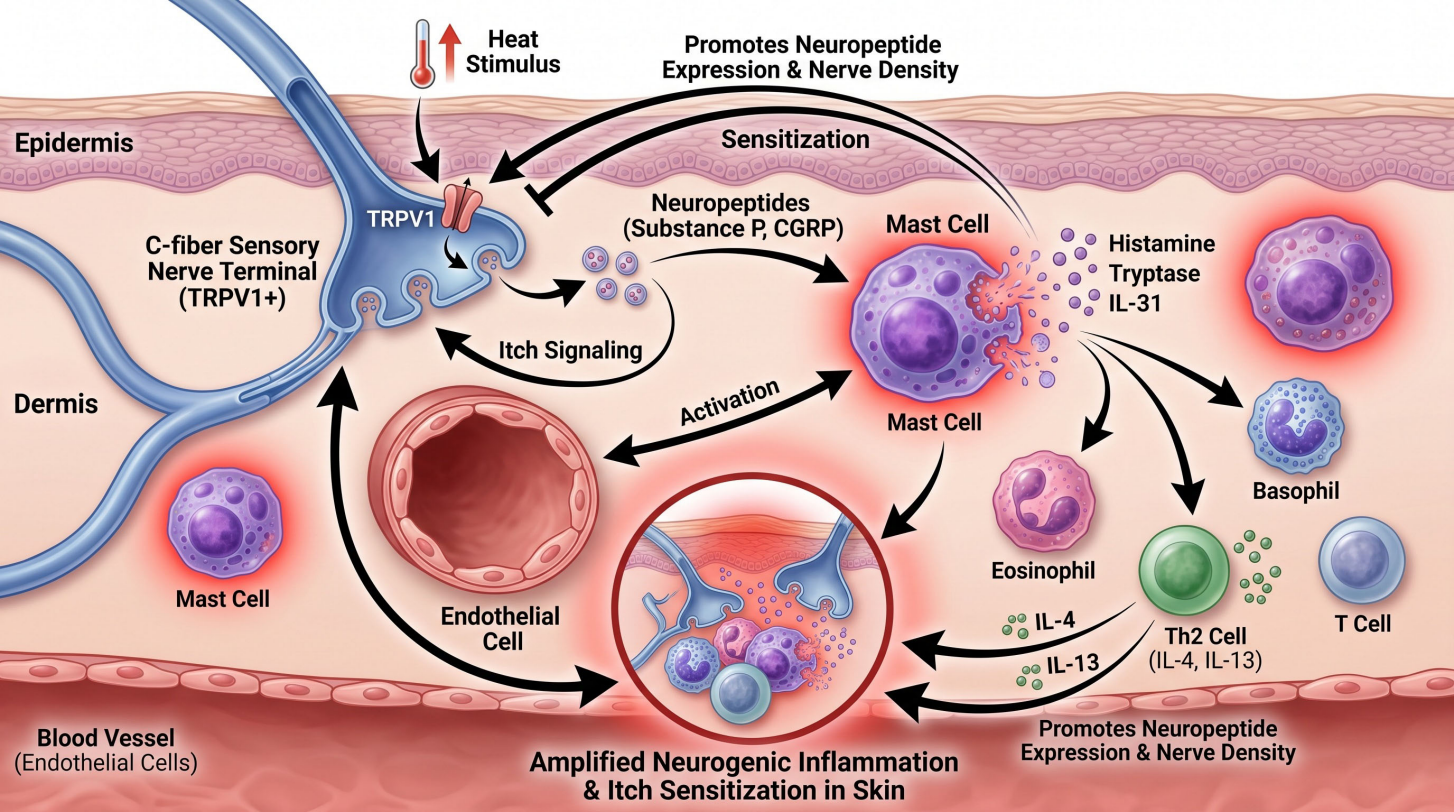
Regulation of neuro-immune interaction network.

The neuroimmune interaction in chronic spontaneous urticaria (CSU) is profoundly bidirectional, with activated immune cells providing critical feedback that sensitizes the nervous system, thereby lowering the threshold for symptom provocation and perpetuating disease chronicity. Mast cells (MCs), once activated via IgE-dependent or MRGPRX2-mediated pathways, release a plethora of mediators that directly act on adjacent sensory nerve endings. Key among these are histamine, tryptase, and interleukin-31 (IL-31) (47). Histamine, acting on histamine H1 receptors on neurons, and tryptase, activating protease-activated receptors (PARs), enhance neuronal excitability and contribute to itch sensation (47). Notably, the antihistamine mepyramine has been shown to directly inhibit voltage-gated sodium channels on nociceptors, which may contribute to its effects beyond H1 receptor blockade, illustrating the direct pharmacological interface between mast cell mediators and neuronal signaling (48). IL-31, a potent pruritogenic cytokine, is particularly significant; its levels are elevated in CSU and it acts on the IL-31 receptor complex (IL-31RA) expressed on sensory neurons to directly induce and potentiate itch (46, 47). This creates a feed-forward loop where MC-derived IL-31 sensitizes nerves (84), leading to increased scratching and potentially further MC activation. Furthermore, infiltrating T helper 2 (Th2) cells secrete cytokines such as IL-4 and IL-13 also induces pruritus and neuronal sensitization (102, 103), which can modulate the local neural environment by regulating the expression of neuropeptides and influencing nerve fiber density and sprouting (45). This immune-mediated neuroplasticity strengthens the coupling between nerves and immune cells. The consequence of this bidirectional crosstalk is a state of peripheral sensitization, where even minor fluctuations in body temperature or subtle psychological stressors can trigger a disproportionate release of neuropeptides and MC mediators, leading to intense pruritus and whealing (49, 50). Psychological stress, a common comorbidity in CSU, exacerbates this cycle by dysregulating the hypothalamic-pituitary-adrenal (HPA) axis, further priming MCs and sensitizing sensory nerves, thus embedding the disease within a bio-psycho-social framework (45, 49). This core mechanism of immune-to-nerve feedback, characterized by the lowering of neuronal activation thresholds and the enhancement of neurogenic inflammation, explains the recurrent and often refractory nature of CSU symptoms, where the nervous system becomes an amplifier of immune dysregulation (**Figure 2**).

## 5 Coupling mechanisms of thermoregulation and inflammatory response

### 5.1 Biological basis of body temperature as a physical trigger

The elevation of core body temperature, a cardinal feature in the pathogenesis of cholinergic urticaria (CholU), serves as the fundamental physical trigger by inducing a cascade of physiological changes in the skin’s microenvironment. This increase in temperature, typically resulting from exercise, passive warming, or emotional stress, directly impacts cutaneous microcirculation and eccrine sweat gland activity (51, 58). The resultant sweating is not merely a thermoregulatory response but a critical event that alters the local milieu, potentially modifying factors such as pH and osmotic pressure. These alterations can subsequently influence the functional state of resident immune cells, notably mast cells, and cutaneous sensory nerve endings, setting the stage for symptom generation (59). Crucially, the rise in temperature itself acts as a direct activator of specific ion channels, such as Transient Receptor Potential Vanilloid 1 (TRPV1), which are expressed on cutaneous sensory neurons. The activation of these channels by thermal stimuli is hypothesized to be a primary “trigger” that initiates afferent neural signaling, thereby engaging the neuro-immune axis central to CholU pathophysiology (60). Furthermore, exercise-induced hyperthermia is often accompanied by the release of endogenous opioid peptides. These neuropeptides may modulate immune cell activity and pruritic or painful sensations through complex, yet not fully elucidated, mechanisms, adding another layer to the neurogenic component of the disorder (16). The clinical link between temperature elevation and symptom onset is robust, with provocation tests using exercise or controlled warming reliably inducing the characteristic pinpoint wheals and associated pruritus or stinging pain in affected individuals (13, 61). This intimate connection underscores that the biological response to increased core temperature is not a passive background event but an active driver of the pathological process, integrating vascular, glandular, neural, and immune components to manifest the clinical phenotype of CholU.

### 5.2 The non-classical role of the cholinergic system

The historical nomenclature “cholinergic urticaria” implies a central pathogenic role for acetylcholine (ACh), yet contemporary evidence increasingly suggests its role is limited and that the condition is mediated by broader neuro-immune interactions. Traditionally, ACh released from sudomotor nerve endings upon thermoregulatory stimulation was considered the primary mediator inducing mast cell degranulation. However, this paradigm is challenged by the generally poor efficacy of anticholinergic medications in treating CholU (17). Intradermal testing with ACh in patients yields positive wheal-and-flare responses in only a subset of individuals. While a positive ACh skin test lacks diagnostic sensitivity, the finding of heightened responsiveness in CholU patients is not contradictory (62).

While we contend that ACh is not the primary initiator, it would be an oversimplification to entirely dismiss its potential role in disease amplification. The chronic inflammatory environment in CholU (rich in histamine, tryptase, and IL-31) may induce a state of non-specific neuronal hyperexcitability, affecting both sensory and autonomic nerves. Autonomic nerve endings might become hypersensitive due to chronic exposure to the inflammatory soup in the skin of CholU patients. This creates permissive conditions where typically sub-threshold stimuli (heat, ACh) can trigger symptoms.

The systemic cholinergic anti-inflammatory pathway, mediated by vagal nerve release of ACh acting on the α7 nicotinic ACh receptor (α7nAChR) on macrophages, is a well-established regulator of systemic inflammation. However, its role in the cutaneous compartment during CholU appears to be either dysregulated or functionally minimal, failing to counteract the local inflammatory cascade effectively (63). In fact, investigations into sweat gland physiology in CholU patients with impaired sweating have revealed reduced expression of the muscarinic acetylcholine receptor M3 (CHRM3) and acetylcholine esterase in the sweat gland epithelium, suggesting a possible defect in cholinergic signaling at the effector organ level rather than an excess of cholinergic stimulation (63). The “cholinergic” component may not be the primary driver but could act as a pathological amplifier within a dysregulated neuro-immune network. Furthermore, studies in heart donors with induced long QT syndrome have shown neuro-immune dysregulation, altered immune profiles with T cell activation and Th2 enrichment, suggesting a failure in immune regulation that could be analogous to pathways in urticaria (56).

The core pathophysiology has expanded to encompass a complex interplay involving direct thermal activation of sensory neurons (e.g., via TRPV1), sweat gland dysfunction, possible hypersensitivity to sweat components themselves (sweat allergy), and the pivotal degranulation of skin mast cells with release of histamine and other mediators like tryptase (18, 60, 64). This shift in understanding refocuses the disease model from a purely cholinergic phenomenon to a disorder of aberrant neuro-immune crosstalk, where the initial thermal/neural signal converges with immune effector cell activation in the skin to produce symptoms.

## 6 Imbalance of adaptive immunity and cytokine network

### 6.1 Dominant role of Th2 immune deviation

A Th2-type immune deviation is a central feature in the pathogenesis of chronic spontaneous and cholinergic urticaria (CSU/CholU), characterized by a predominant Th2 cell response in both lesional skin and the systemic environment. This skewing is marked by elevated levels of signature Th2 cytokines, including interleukin-4 (IL-4), interleukin-13 (IL-13) (51). The critical roles of IL-4 and IL-13 are multifaceted. They promote B cell class-switching to produce immunoglobulin E (IgE) and potentially pathogenic autoantibodies, thereby priming the mast cell (MC) and basophil degranulation pathway central to urticaria (51). Furthermore, these cytokines enhance vascular endothelial cell reactivity to inflammatory mediators, contributing to vasodilation, plasma extravasation, and wheal formation (52). IL-31, identified as a potent pruritogen, directly links immune activation to the hallmark symptom of itching in urticaria (46). The IL-33-ST2 axis in memory Th2 cells has been shown to control peripheral sensory C-fiber axonal elongation and the induction of severe itch, with calcitonin gene-related peptide (CGRP) produced by these pathogenic Th2 cells cooperating with somatosensory neurons to drive pruritus (53). This neuro-immune crosstalk underscores IL-31 and related pathways as crucial therapeutic targets. The clinical efficacy of biologics targeting the Th2 axis supports its dominance; for instance, dupilumab, a monoclonal antibody that inhibits IL-4 and IL-13 signaling, has demonstrated success in inducing remission of CSU and controlling cholinergic flare-ups in case reports (11, 51). Its mechanism is proposed to involve blocking the IL-4 pathway, reducing the expression of the high-affinity IgE receptor (FcϵRI) on B cells, mast cells, and basophils, thereby decreasing IgE-mediated MC activation and histamine release (51). Data from phase 3 of the study demonstrated that dupilumab improved clinical symptoms such as pruritus and reduced urticaria activity in CSU patients who remained uncontrolled despite H1-AH therapy. However, its response was limited in the specific population of Omalizumab non-responders. We hypothesize that this group often has autoimmune CSU (e.g., anti−FcϵRI antibodies) that directly activate mast cells without requiring IgE or IL−4/IL−13 amplification (54). The Th2 immune deviation, through its characteristic cytokines, not only drives IgE-mediated hypersensitivity and vascular changes but also directly engages the sensory nervous system to produce symptoms, solidifying its central role in CSU/CholU pathophysiology.

### 6.2 Involvement of other immune cells

Beyond mast cells and Th2 lymphocytes, other immune cells contribute to the complex immunopathology of chronic spontaneous and cholinergic urticaria, modulating inflammation, symptom initiation, and chronicity. Basophils serve as an alternative source of histamine and other mediators. Their function and reactivity appear enhanced in some patients, potentially participating in disease initiation (55). Decreased peripheral basophil counts are relatively common in clinical practice. This phenomenon may be due to the recruitment of basophils to the site of skin lesions in CSU (104). Basophils are recognized for their ability to secrete IL-4 and other type 2 cytokines, which can amplify Th2 responses (55). Emerging evidence also suggests basophils can regulate inflammation by engaging in neuro-immune interactions, a function that may be relevant in the context of urticaria where neural triggers are common (55). Although their role is less defined than in other allergic diseases. Once activated, eosinophils release cytotoxic granule proteins (e.g., major basic protein) and cytokines that can exacerbate tissue damage, perpetuate chronic inflammation, and potentially contribute to the late-phase or chronic aspects of the urticarial response (52). A relative insufficiency or functional impairment of regulatory T cells (Tregs) is another proposed component. Tregs are essential for maintaining immune tolerance and suppressing aberrant immune responses against self-antigens or harmless allergens. In CSU/CholU, an inability of Tregs to effectively inhibit abnormal immune reactions against autoantigens or other endogenous triggers may allow for the uncontrolled activation of mast cells and basophils (56). Furthermore, neuro-immune interactions involving innate lymphoid cells type 2 (ILC2s) have been documented in allergic asthma models, where neuropeptides like CGRP can activate ILC2s to release Th2 cytokines, creating a positive feedback loop that exacerbates inflammation (57). While not yet fully elucidated in urticaria, such mechanisms highlight how dysregulation across multiple immune cell types—basophils, eosinophils, Tregs, and potentially ILC2s—interacts with neuronal signals to sustain the chronic inflammatory state and symptom burden in CSU/CholU.

## 7 Efficacy evaluation and limitations of existing treatment strategies

### 7.1 Standard dose and up-dosed antihistamines

Second-generation non-sedating H1-antihistamines (sgAHs) are universally endorsed as the first-line therapy for chronic spontaneous urticaria (CSU), including its cholinergic variant (65). However, a significant therapeutic gap exists, as only approximately 50% of patients achieve adequate symptom control with standard licensed doses (66). This limited efficacy underscores that histamine is not the sole mediator driving the disease, particularly in cholinergic urticaria where neurogenic and other inflammatory pathways are implicated. International guidelines, therefore, recommend a stepwise approach, advising an increase in the antihistamine dose up to fourfold the standard dose for patients who remain symptomatic (65). This recommendation for up-dosing is supported by real-world evidence showing that a substantial proportion of patients who do not respond to standard doses can achieve remission with escalated therapy (67). For instance, studies indicate that up-dosing sgAHs can provide remission in approximately 38.3% of patients who were previously uncontrolled (67). The mechanism behind the benefit of higher doses likely extends beyond simple H1-receptor blockade. Antihistamines possess additional properties, including anti-inflammatory effects and membrane stabilization, which may become more clinically relevant at elevated doses and contribute to controlling the complex pathophysiology of CSU (68). This is corroborated by studies showing that treatment with H1-antihistamines can normalize altered systemic markers like thiol-disulfide homeostasis parameters, which are involved in oxidative stress defense, suggesting a broader modulatory effect (68).

The safety profile of up-dosed second-generation antihistamines is generally favorable, which supports their use in clinical practice. Reviews of specific agents like bilastine, levocetirizine, fexofenadine, and cetirizine indicate they can be safely up-dosed without a dose-dependent increase in adverse effects, and there are no documented reports of systemic complications such as cardiotoxicity at these higher-than-licensed doses (69). For example, bilastine has been shown to be effective and well-tolerated when up-dosed to 40 mg or even 80 mg daily in patients uncontrolled on other sgAHs (70). Similarly, a study on sleep patterns in CSU patients found that up-dosing sgAHs improved objective sleep measures like REM sleep without increasing daytime sleepiness, highlighting a good neurological tolerability profile (71). However, the evidence base is heterogeneous, and some older systematic reviews note the low quality of many studies and the need for larger, robust randomized controlled trials to solidify these recommendations (72). Despite the overall safety, vigilance is required, particularly regarding long-term use in specific populations. Data on the safety of up-dosing in geriatric patients, pregnant women, and lactating females are scarce (69). Furthermore, while not commonly reported, the potential for cardiac effects, especially with certain antihistamines or in patients with predisposing conditions, necessitates ongoing monitoring (73). The clinical decision to up-dose must therefore balance the proven efficacy and general safety with the acknowledgment that prolonged ineffective antihistamine therapy should be avoided to prevent delayed disease control and unnecessary socioeconomic burden (74).

### 7.2 Early application of immunomodulators and biologics

For patients with chronic spontaneous urticaria (CSU) who remain symptomatic despite optimized antihistamine therapy, including up-dosing, the early introduction of immunomodulators and biologics is a critical step. Omalizumab, a monoclonal anti-IgE antibody, represents the cornerstone of second-line biologic therapy and has demonstrated significant efficacy in antihistamine-refractory CSU, including cases with cholinergic symptoms (31). Its mechanism involves reducing free IgE levels and downregulating the expression of the high-affinity IgE receptor (FcϵRI) on mast cells and basophils, thereby attenuating their activation (39). Real-world and clinical trial data confirm its effectiveness; for instance, omalizumab monotherapy was effective in 66.9% of patients in one real-life study, with treatment response significantly better in patients with higher total IgE levels (75). Furthermore, studies have explored optimizing omalizumab use, showing that in patients with well-controlled disease on omalizumab, as-needed sgAHs can still effectively manage intermittent breakthrough symptoms (76). The success of omalizumab firmly establishes the centrality of the IgE-mediated pathway in a substantial subset of CSU patients. However, a significant limitation is that approximately 30% or more of patients exhibit an insufficient response to omalizumab, highlighting the disease’s heterogeneity and the existence of other driving mechanisms (31). Predictors of poor response to omalizumab include advanced age, high BMI, comorbid autoimmune diseases, low total IgE levels (e.g., <40–50 IU/mL), and positivity for autoantibodies such as ANA or anti-TPO (39).

In cases refractory to both antihistamines and omalizumab, conventional immunomodulators like cyclosporine A are employed as third-line options. Cyclosporine A acts primarily by inhibiting T-cell activation and cytokine production, which is beneficial, particularly in patients with autoimmune features (31). It can improve symptoms in approximately 54% to 73% of patients but is limited by potential adverse effects such as nephrotoxicity and hypertension, requiring close monitoring and often restricting its long-term use (31). The exploration of novel, targeted agents is actively addressing the unmet need in refractory CSU. Bruton’s tyrosine kinase (BTK) inhibitors, which are crucial for FcϵRI-mediated mast cell activation and B-cell autoantibody production, have shown great promise. Fenebrutinib, a reversible BTK inhibitor, demonstrated dose-dependent efficacy in a phase 2 trial for antihistamine-refractory CSU, including in patients with type IIb autoimmunity (77). FDA approval remibrutinib (another oral covalent BTK) was based on Phase III REMIX-1 and REMIX-2 trials, which demonstrated rapid, significant, and sustained improvements in Urticaria Activity Score (UAS7), Itch Severity Score (ISS7), and Hives Severity Score (HSS7) compared with placebo (78). The recent phase 2 RILECSU trial further confirmed the efficacy of the BTK inhibitor rilzabrutinib at a dose of 1200 mg/day, showing significant improvements in itch and hive scores as early as week 1 and reductions in CSU-related biomarkers (79). Other innovative approaches include mast cell-targeted therapies. Barzolvolimab, a monoclonal anti-KIT antibody that depletes mast cells, has shown rapid and profound symptom reduction in phase 1b and 2 trials for antihistamine-refractory CSU, providing direct evidence for the essential role of mast cells in driving clinical manifestations (80, 81). In addition to barzolvolimab, other KIT-targeting monoclonal antibodies (e.g., CDX-0159) are under investigation (85).

## 8 Emerging targeted therapies and future research directions

### 8.1 Biologics targeting specific cytokines

The development of biologics targeting specific cytokine pathways represents a significant advancement in the management of chronic spontaneous urticaria (CSU), particularly for patients who are refractory to conventional therapies like H1-antihistamines and omalizumab (85). Among these, dupilumab, a fully human monoclonal antibody that inhibits the interleukin-4 receptor alpha (IL-4Rα), thereby blocking the signaling of IL-4 and IL-13, has shown considerable promise. These cytokines are central to the Th2 immune response, which is increasingly recognized as a key pathogenic driver in CSU (86). While dupilumab is approved for conditions like atopic dermatitis (AD), asthma, and chronic rhinosinusitis with nasal polyps (CRSwNP), emerging evidence from case reports and small studies suggests its potential efficacy in refractory CSU (51). For instance, a case report documented the successful off-label use of dupilumab in a patient with cholinergic urticaria, leading to the cessation of symptoms and a marked improvement in quality of life, potentially through the downregulation of the high-affinity IgE receptor (FcϵRI) on mast cells and basophils (51). Furthermore, the co-existence of CSU with other Th2-mediated conditions like AD, which share overlapping immunopathogenic mechanisms, provides a rationale for using dupilumab in patients with such comorbidities (88). As research progresses, dupilumab has now received CSU approval in multiple regions. Beyond IL-4/IL-13, other cytokine targets are under investigation. For example, the anti-IL-5 receptor-α monoclonal antibody benralizumab, which depletes eosinophils, did not demonstrate clinical benefit over placebo in a phase IIb trial despite achieving near-complete blood eosinophil depletion, suggesting eosinophils may not be a primary effector cell in all CSU endotypes (82). Similarly, the IL-1β antagonist canakinumab was not effective in a phase II study of moderate-to-severe CSU, indicating that the IL-1 pathway may not be crucial in typical CSU pathology (83). Antibodies targeting the IL-31 receptor are being developed primarily for pruritic diseases, with the potential to directly interrupt the itch-scratch cycle, a major component of CSU that severely impacts patient quality of life (86). Additionally, biologics directed against epithelial cell-derived alarmins, such as thymic stromal lymphopoietin (TSLP), aim to intervene at the very initiation of the inflammatory cascade. Tezepelumab, a monoclonal antibody that inhibits TSLP, represents a novel approach that may address both type 2 and non-type 2 inflammation, broadening the therapeutic scope (87, 89). Regrettably, the Phase 2b study was declared a failure. The exploration of these targeted biologics is part of a broader, promising pipeline for CSU that aims to address the heterogeneous pathomechanisms of the disease, moving towards more personalized treatment strategies (85, 90). As research progresses, integrating cytokine profiling with clinical phenotypes will be crucial for refining patient stratification and optimizing the deployment of these advanced therapies (86).

### 8.2 Targeted intervention at neuro-immune interaction nodes

Targeting the intricate crosstalk between the nervous and immune systems presents a novel frontier for therapeutic innovation in chronic spontaneous urticaria (CSU) and related conditions (91). One strategic approach involves the development of neurokinin-1 receptor (NK-1R) antagonists designed to block the effects of substance P (SP), a neuropeptide implicated in neurogenic inflammation and pruritus (92). SP, signaling primarily through NK1R, influences immune cell activity and vascular permeability; however, clinical trials of NK-1R antagonists in urticaria have yielded mixed results, underscoring the need to identify patient subgroups where this pathway is predominant (91). Another promising node for intervention is the family of sensory nerve ion channels, particularly the transient receptor potential vanilloid 1 (TRPV1) channel, which is activated by heat and various inflammatory mediators (93). TRPV1 antagonists could theoretically reduce the neural signal transmission triggered by thermal stimuli, a common provocation in cholinergic urticaria, but their development must carefully balance efficacy with the potential disruption of physiological thermosensation and pain perception (93). Furthermore, targeting non-IgE receptors on mast cells offers a pathway for precise intervention without compromising normal IgE-mediated host defense. The Mas-related G protein-coupled receptor X2 (MRGPRX2) has garnered attention as an alternative activation pathway for mast cells, and drugs designed to modulate this receptor could provide a new therapeutic avenue (90). The broader context of neuro-immune communication is increasingly recognized across inflammatory diseases, where sympathetic nerves, macrophages, and cytokines form a dynamic regulatory network (94). In CSU, dysregulation of such neuro-immune-endocrine axes may contribute to chronicity and treatment resistance (95). Therefore, therapeutic strategies that restore balanced neuro-immune crosstalk, potentially through neuromodulation or receptor-specific agents, hold significant promise. These approaches aim to move beyond broad immunosuppression towards mechanisms that specifically dampen the pathological dialogue between sensory neurons, immune cells like mast cells and basophils, and the inflammatory mediators they release, thereby addressing both the wheals and the debilitating pruritus characteristic of CSU (91).

### 8.3 Personalized medicine and disease endotype subdivision

**Figure 3:**
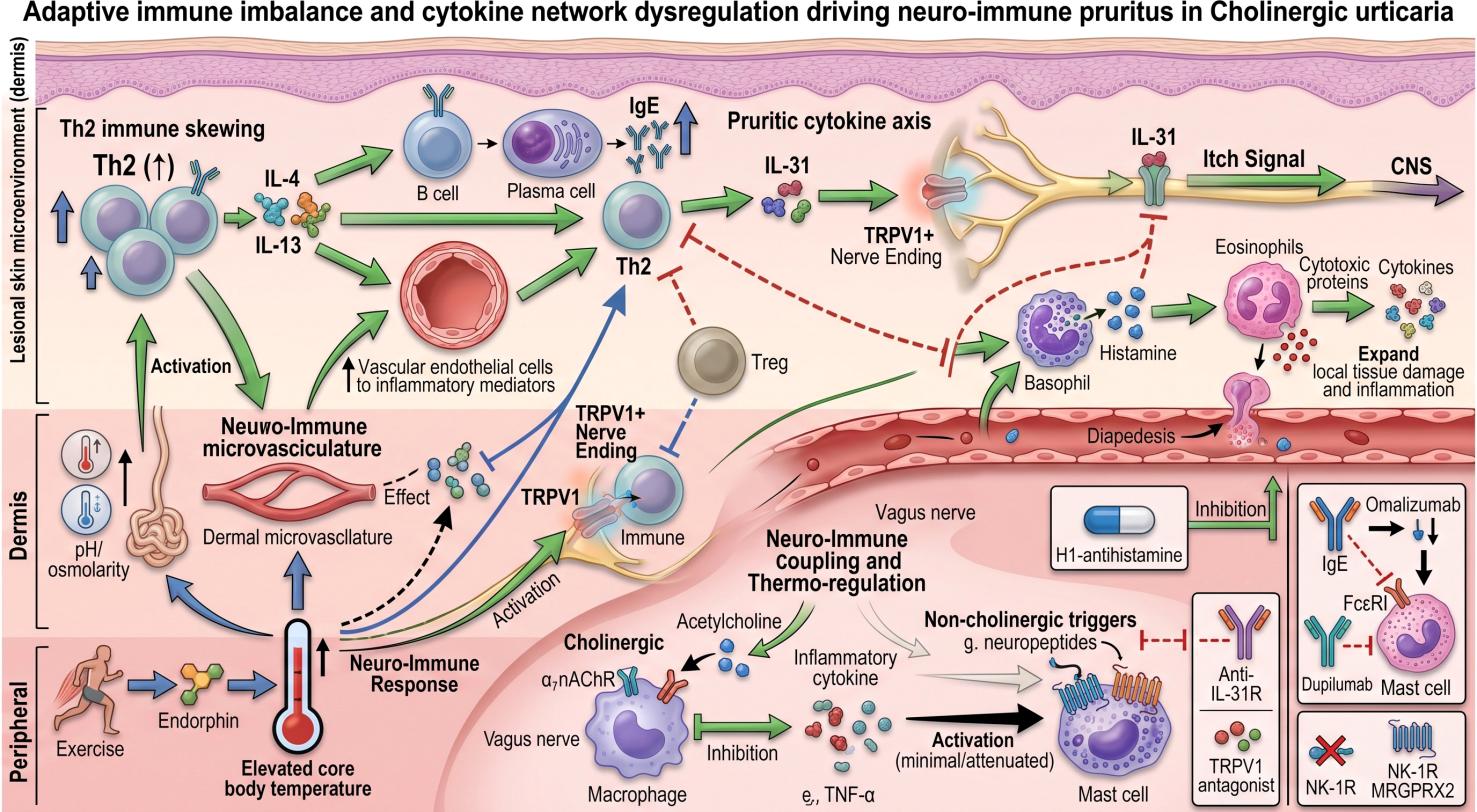
In cholinergic urticaria, the abnormal activation of the immune system (especially the Th2 pathway) interacts with nerve receptors (TRPV1+) to jointly cause persistent itching. Existing therapies intervene in multiple targets of the immune or nervous system to alleviate symptoms.

The future of chronic spontaneous urticaria (CSU) management lies in personalized medicine, which necessitates a deep understanding of disease heterogeneity through the identification of distinct endotypes based on underlying biological mechanisms (90). A critical step towards this goal is the discovery and validation of reliable biomarkers that can stratify patients into clinically relevant subgroups. Potential biomarkers include specific autoantibodies (e.g., against IgE or FcϵRI), serum cytokine profiles (e.g., levels of IL-4, IL-13, IL-31, IL-33, or IL-17), and neuropeptide concentrations, which reflect different pathogenic drivers such as autoimmunity, predominant Th2 inflammation, or neuro-immune dysregulation (96, 97). For example, baseline serum levels of IL-2, IL-13, IL-31, and IL-33 have been identified as potential predictors of response to omalizumab, highlighting the utility of cytokine profiling in guiding therapy (96). To comprehensively dissect this heterogeneity, the integration of multi-omics technologies—including genomics, transcriptomics, proteomics, and metabolomics—is essential (98). These approaches can uncover novel driver genes, signaling pathways, and molecular networks specific to different CSU endotypes, moving beyond the current clinical classifications to a mechanism-based understanding of the disease (99). Furthermore, the development of sophisticated experimental models that accurately simulate CSU pathophysiology is crucial for accelerating drug discovery and validation. This includes advanced *in vitro* models using patient-derived cells (e.g., mast cells, basophils) to study degranulation and cytokine release, as well as animal models that recapitulate key features of human CSU, such as spontaneous wheal formation and response to neuro-immune triggers (100). The ultimate aim is to match each patient’s unique biological signature with the most appropriate targeted therapy, whether it be an anti-IgE biologic, an anti-cytokine agent like dupilumab or tezepelumab, a Bruton’s tyrosine kinase (BTK) inhibitor, or a neuro-immune modulator (85, 101). This precision medicine paradigm, fueled by biomarker discovery, multi-omics profiling, and robust preclinical models, promises to transform CSU care by improving treatment efficacy, reducing trial-and-error prescribing, and ultimately achieving better long-term outcomes for patients (90, 99) (**Figure 3**).

## 9 Conclusion

Chronic spontaneous cholinergic urticaria (CSU-Chol) represents a paradigm of neuro-immune dysregulation, where the central role of mast cell aberrant activation is sustained by an intricate pathological background of autoimmune reactivity and Th2-skewed inflammation. The evolution of our understanding from a purely histamine-centric model to a systems-based network disorder underscores a significant shift in both conceptual and therapeutic frameworks. This review has delineated how core temperature elevation acts not merely as a physical trigger but as a critical initiator of a self-perpetuating vicious cycle, wherein sensory neuron-derived neuropeptides engage in a maladaptive crosstalk with the immune compartment. The limitations of conventional H1-antihistamines, while providing symptomatic relief for many, have starkly highlighted the unmet need in a substantial patient subset, thereby catalyzing the exploration of targeted immunomodulation. The transformative efficacy of biologics directed against IgE (e.g., omalizumab) and the IL-4/IL-13 axis (e.g., dupilumab) has not only validated the pathogenic relevance of these pathways but has also firmly established immune-targeted strategies as a cornerstone of modern management, moving the field beyond mere symptom suppression towards modifying the underlying disease process.

From an expert perspective, the current landscape of CSU-Chol research and therapy is characterized by a dynamic tension between holistic network understanding and reductionist target pursuit. The central challenge lies in balancing the recognition of the disease’s inherent complexity—where neural signals, autoimmune antibodies, and inflammatory cytokines converge on the mast cell—with the pragmatic necessity of identifying discrete, druggable nodes within this network. The success of biologics exemplifies the power of the latter approach, yet their variable response rates remind us that the former, more integrative view is essential for further progress. For instance, the efficacy of omalizumab, even in some patients without clearly elevated IgE, suggests its action may extend beyond simple IgE neutralization to modulating mast cell releasability and FcϵRI expression, effects that resonate within the broader neuro-immune context. Similarly, the exploration of dupilumab, a Th2 pathway inhibitor, in a condition with a cholinergic trigger, beautifully illustrates the convergence of disparate research perspectives: it targets the sustained inflammatory milieu that likely lowers the threshold for neuronally-mediated mast cell degranulation.

The future trajectory of CSU-Chol treatment hinges on deepening our dissection of the specific molecular dialogues within the neuro-immune synapse. Promising frontiers include the development of precision therapeutics aimed at neuropeptide receptors (e.g., antagonists for substance P or calcitonin gene-related peptide), components of the specific itch-signaling pathways (targeting mediators like IL-31 or neural receptors such as MrgprX2), and novel mast cell activation pathways beyond IgE/FcϵRI. However, the ultimate translation of these discoveries into clinical benefit will be critically dependent on our ability to deconstruct the apparent homogeneity of CSU-Chol into distinct endophenotypes. The goal is to move from a trial-and-error treatment paradigm to a predictive, biomarker-guided strategy. Identifying biomarkers—whether serological (e.g., specific autoantibodies, cytokine profiles), cellular, or even based on provocation test characteristics—will enable the stratification of patients into subgroups more likely to respond to a given mechanism-targeted therapy. This could mean distinguishing patients where the autoimmune component is dominant from those where neural dysregulation is primary, or identifying those with a prominent IL-4/IL-13 signature versus other inflammatory patterns.

Therefore, the path forward requires a synergistic dual approach: continued investment in basic science to unravel the fundamental mechanisms and identify novel targets, coupled with robust clinical research focused on biomarker discovery and validation in well-phenotyped patient cohorts. The integration of multi-omics technologies, advanced neuroimaging, and detailed clinical phenotyping will be instrumental. The overarching objective is to achieve true personalized medicine, where the most effective and safest therapeutic agent is matched to the individual patient’s unique disease drivers. This precision not only promises superior short-term symptom control but also holds the potential to alter the long-term disease course, prevent chronicity, and fundamentally improve the quality of life for patients burdened by this complex and distressing condition. In conclusion, CSU-Chol stands as a compelling model of how bridging neurobiology and immunology can illuminate disease pathogenesis and catalyze therapeutic innovation, setting a precedent for other chronic inflammatory disorders characterized by similar neuro-immune interplay.
